# Application of the Theoretical Domains Framework and the Behaviour Change Wheel to Understand Physicians’ Behaviors and Behavior Change in Using Temporary Work Modifications for Return to Work: A Qualitative Study

**DOI:** 10.1007/s10926-017-9706-1

**Published:** 2017-04-08

**Authors:** Ritva Horppu, K. P. Martimo, E. MacEachen, T. Lallukka, E. Viikari-Juntura

**Affiliations:** 1 Work Disability Prevention, Finnish Institute of Occupational Health, P.O. Box 40, 00251 Helsinki, Finland; 2 School of Public Health and Health Systems, University of Waterloo, Waterloo, Canada; 3 Centre for Research on Work Disability Policy, Institute for Work & Health, Toronto, Canada

**Keywords:** Behavior change, Physicians, Occupational health services, Return to work

## Abstract

*Purpose* Applying the theoretical domains framework (TDF) and the Behaviour Change Wheel (BCW) to understand physicians’ behaviors and behavior change in using temporary work modifications (TWMs) for return to work (RTW). *Methods* Interviews and focus group discussions were conducted with 15 occupational physicians (OPs). Responses were coded using the TDF and the BCW. *Results* Key behaviors related to applying TWMs were initiating the process with the employee, making recommendations to the workplace, and following up the process. OP behaviors were influenced by several factors related to personal capability and motivation, and opportunities provided by the physical and social environment. Capability comprised relevant knowledge and skills related to applying TWMs, remembering to initiate TWMS and monitor the process, and being accustomed to reflective practice. Opportunity comprised physical resources (e.g., time, predefined procedures, and availability of modified work at companies), and social pressure from stakeholders. Motivation comprised conceptions of a proper OP role, confidence to carry out TWMs, personal RTW-related goals, beliefs about the outcomes of one’s actions, feedback received from earlier cases, and feelings related to applying TWMs. OPs’ perceived means to target these identified factors were linked to the following BCW intervention functions: education, training, persuasion, environmental restructuring, and enablement. The results suggest that at least these functions should be considered when designing future interventions. *Conclusions* Our study illustrates how theoretical frameworks TDF and BCW can be utilized in a RTW context to understand which determinants of physicians’ behavior need to be targeted, and how, to promote desired behaviors.

**Electronic supplementary material:**

The online version of this article (doi:10.1007/s10926-017-9706-1) contains supplementary material, which is available to authorized users.

## Introduction

Daily activities (incl. appropriate work) are beneficial for recovery, especially from musculoskeletal and mental disorders [1]. Therefore, staying at work (SAW) or returning to work (RTW) after short absence from work has become advisable. In many countries, the legislation has been amended in order to increase possibilities for work task and work time arrangements, such as the use of part-time sick leave [2], the introduction of the fit note and the development of national return-to-work programs [3–5]. Work accommodations can include modified or alternate duties, workstation redesign, activity restrictions, reduced hours, or other efforts to temporarily reduce physical or mental work demands. In our earlier qualitative study, occupational physicians considered SAW/RTW with temporary work modifications (TWMs) to a large extent beneficial for employees and other stakeholders if the RTW situation and context are taken into consideration [6].

Previous studies have identified various barriers to and enablers of using TWMs, as reported by physicians using the fit note [7–10] or supporting RTW more generally [11–14]. Physicians have described challenges related to personal knowledge, skills, and conceptions of a physician’s role, as well as to environmental factors, such as hesitancy or negative attitudes of various workplace stakeholders, and scarcity of clear RTW procedures at workplaces, such as lack of accommodated work; and the level of cooperation with all parties. Occupational physicians (OPs) do not generally provide medical treatment to employees. Not being involved in the RTW process from the beginning and not having easy access to employees’ health status have also been reported as barriers of applying TWMs [15].

Few studies have utilized theoretical models or frameworks to understand the factors influencing practitioners’ behavior in using work modifications. Van Duijn et al. [13] applied a health education model in investigating physicians’ conceptions of the barriers to implementing modified work in companies. Fassier et al. [11] developed a conceptual framework for identifying practitioners’ perceptions of the factors influencing the implementation of a workplace-based RTW program. However, these studies do not provide theoretically informed means for addressing the recognized implementation problems.

We utilized two frameworks, the Behaviour Change Wheel (BCW) and the Theoretical Domains Framework (TDF), which together provide a comprehensive assessment of the factors that are likely to influence practitioners’ target behavior and a theoretically informed development of interventions to promote the desired behavior. The BCW is a synthesis of 19 theoretical frameworks of behavior change [16, 17] and is based on a model of human behavior, the COM-B model, which presents human behavior (B) as resulting from interaction between physical and psychological capabilities (C), opportunities provided by the physical and social environment (O), and reflective and automatic motivation (M).

The BCW also presents component-specific intervention functions with enabling policy categories to promote the desired behavior. For example, in order to influence capability, the following intervention functions could be considered: education, training, modeling, enablement, or environmental restructuring. Furthermore, various behavior change techniques can be utilized to serve each intervention function [17, 18]. For example, education and training can include specific techniques such as delivering information, teaching skills and supporting practitioners’ own behavior regulation (e.g., goal setting, self-monitoring, and action planning).

The TDF has been developed and validated for behavior change and implementation research [19, 20], and can be used for a detailed analysis of the potentially modifiable factors (falling under the three COM-B components) to target in an intervention. The refined TDF is composed of 84 constructs from multiple psychological theories (motivational, action, and organizational theories) and consists of 14 domains of theoretical constructs.

The BCW and/or the TDF have been used to investigate factors influencing physicians’ behavior and to design interventions in various clinical contexts, for example, primary care, hospital and other care facilities [21–28]. We are not aware of research using these frameworks in the RTW context or in the context of occupational health more generally.

Utilizing theoretical frameworks (the TDF and the BCW), the aims of our study were

1. to identify factors that are likely to influence OPs’ behaviors related to applying TWMs and that could be targeted in future interventions; and
2. to evaluate the possible applicability of the intervention functions proposed by the BCW by investigating physicians’ perceived means of overcoming the barriers and/or enhancing the enablers.

Finnish OPs usually provide a general practice level of medical care in addition to the preventive occupational health services. This, together with OPs’ duty to encourage RTW, creates an ideal opportunity to initiate temporary work accommodations.

## Methods

This study is part of a larger educational intervention project aimed at enhancing SAW and early RTW via OPs’ increased deliberation, recommendations, and guidance about temporary work modifications among employees with musculoskeletal pain or depressive symptoms (ISRCTN74743666).

## Participants and Procedure

First, pilot interviews were conducted with four Finnish OPs in order to form ideas for focus group questions with OPs. A purposive sampling strategy was used to reach participants with variable experience of applying TWMs. Pilot interviews took place at the participants’ workplaces, and they lasted approximately 60 min.

The pilot interviews were guided by a semi-structured topic guide, based on previous scientific literature and discussions among the authors. The domains of the TDF were not considered when designing the interview topic guide. These initial interviews asked questions about the RTW process and OP behaviors related to using TWMs, participants’ perceptions of barriers to and enablers of this practice, and their experiences of and suggestions for how to overcome the barriers and enhance the enablers.

Knowledge developed in the pilot interviews led to foci for discussion in subsequent focus groups [6]:

- usefulness of TWMs
- suitability of TWMs to musculoskeletal and mental disorders
- responsibility of OPs to initiate TWMs
- supervisors’ willingness to implement TWMs
- confidentiality of health-related issues on using TWMs

The main data collection, focus group discussions, followed. Focus groups were chosen as the method because we wanted to engage the participants in explaining and justifying their TWMs-related understanding and behavior to one-another. This approach encouraged them to prompt each other, allowing a deeper insight into their perceptions and justifications of behaviour [29–31]. In these discussions, the participants were challenged to deliberate about the propositions formed from the pilot interviews. They were encouraged to express divergent arguments about encouraging SAW/RTW via TWMs.

In the focus groups, after presenting a proposition the moderator asked participants to state whether they agreed or disagreed with it and to justify their positions. Subsequent questions were posed to follow up the arguments or accounts of experiences. Contrasting perspectives were actively raised to explore participants’ reasoning in more depth. No requests were made to reach a consensus in the discussions. Consistent with the pilot interviews, participants were asked to describe their experiences and perceptions of the barriers to and enablers of applying TWMs as well as their experiences of and suggestions for means to target the identified factors.

A purposive sampling approach was also used to compose focus groups in which participants would be similar enough, but vary sufficiently in order to allow for differences in experiences and perceptions. We aimed to recruit OPs of both genders and of varying years of experience in occupational health (OH). Ten OPs at their later stage in specializing in occupational health at the Finnish Institute of Occupational Health (FIOH) were asked to take part in the interviews. In addition, 20 specialists in OH were approached by e-mail and asked to participate after they had attended a further education course related to mentoring junior OPs. Six physicians in both groups agreed to participate. Non-participation was mainly justified by time constraints.

Four focus groups with 11 OPs in total were conducted. We had two all-female groups and two groups of both genders. Three groups were held with three participants, and one group consisted of two participants only, as one of the enrolled participants was unable to attend. Focus group discussions took place at the FIOH, and lasted approximately 90 min.

The first author served as a moderator in the pilot interviews and focus groups. The moderator is a social psychologist with a long experience in conducting interviews and facilitating focus group discussions. She has also practiced as a pedagogical advisor for developing the specialist training of OPs. Being familiar to this field, but not being an OP herself, the moderator was less likely to influence the way the participants approached the questions in the interviews and focus group discussions.

At the start of interviews and focus groups, general information of the study was given, and it was emphasized that different points of views were appreciated. All participants were first asked to describe a case where they had used TWMs to support SAW/RTW. In the end of each interview and focus group discussion, participants were asked to raise other issues related to SAW/RTW and TWMs that they considered important but which had not been covered so far. All interviews and focus groups were audio recorded and transcribed verbatim. Field notes were written after discussions to capture data on group interaction.

## Analysis

All data were analyzed by qualitative content analysis, using both inductive and deductive approaches [32–34]. First, inductive content analysis was used to identify and categorize the key behaviors that OPs engage in when applying TWMs to support SAW/RTW. Transcripts were read repeatedly to achieve an overall understanding of the data. During a following detailed reading, all accounts of OP behaviors were marked and labeled with preliminary codes. Later, main codes were decided and used to code the data. Finally, some codes were combined, and all codes were grouped into higher-order categories, i.e., the key OP behaviors.

Next, deductive content analysis was conducted to identify the barriers and facilitators that influence each of the key behaviors. The analysis proceeded from a thorough reading of the data and extraction of influencing factors to systematic coding. The coding framework comprised the 14 TDF domains (Table 1):

- Knowledge; Skills; Memory, attention and decision processes; Behavioural regulation (falling under the Capability component of the COM-B model, included in the BCW);
- Environmental context and resources; Social influences (falling under the Opportunities component);
- Social/Professional role and identity; Beliefs about capabilities; Optimism; Beliefs about consequences; Intentions; Goals; Reinforcement; Emotion (falling under the Motivation component).

Special attention was paid to data that did not fit into the existing codes. All data could be classified according to the TDF, and, consequently, no new categories were developed. Finally, inductive content analysis was used to identify and categorize participants’ domain-specific means of targeting the factors influencing the key behaviors. The identified means were further scrutinized in light of the intervention functions proposed by the BCW. All analyses were performed by the first author, and discussed with other members of the research group.

## Ethical Considerations

The study was accepted by the Coordinating Ethics Committee of Helsinki and Uusimaa Hospital District, Finland (approval number 35/13/03/00/2013). The participants were provided with information about the study and advised that all collected data would be confidential and anonymous. Written consent was obtained from the participants for the recording and transcriptions of the interviews and group discussions. The participants were told that they were entitled to request at any point that any of their comments to be erased from the transcripts. Nobody made this kind of request. Confidentiality was maintained in the study’s analytical and presentational practices by using pseudonyms for the participants.

## Results

All 15 participants were accustomed to using work modifications to support RTW. Eight participants, six women and two men, were experienced specialists in OH. Seven participants, five women and two men, were finalizing their specialist training in occupational health. The median of the participants’ job tenures was 14 years (range 4–37 years), consisting of practice for various types of occupational health providers. Two participants had worked in OH <5 years, three participants had 5–10 years of experience in OH, six participants had worked in OH 11–20 years, and four participants had more than 20 years of experience in OH.

Although experienced in using work modifications, some participants described recommending modified work mostly in relation to RTW from prolonged sick leaves, when more complex and long-term modifications are often needed. Temporary work modifications (TWMs) generally suffice to support RTW when the preceding sick leave is short and the disability typically less severe.

Three key behaviors that OPs engage in when using TWMs to support SAW/RTW were: (1) initiating the process during consultation with the employee; (2) making recommendations to the workplace; and (3) following up the work modification process.

Firstly, when an employee meets with the physician to discuss his or her treatment and potential sickness absence, the physician evaluates whether SAW/RTW with TWMs is recommendable to this employee. If so, the physician introduces this option and further discusses its benefits and risks with the employee. Secondly, when an employee and the physician agree on TWMs, this option is introduced to the supervisor. If necessary, its benefits and other reassurances are presented. Thirdly, physicians may monitor whether TWMs are organized at the workplace, according to the previous agreement. They may also check whether modified work supports the employee’s recovery as planned. In contrast to the previous two key OP behaviors, following up the work modifications process was not spontaneously raised by all interviewees or discussed in all focus groups. This observation suggests that all OPs may not routinely monitor the implementation and effects of the work modifications they recommend.

Various barriers and facilitators were identified to influence the above key behaviors. Table 1 presents the general themes of these factors. A detailed presentation of the barriers and facilitators with illustrative quotes from the interviews, is provided in Online Appendix A.

**Table 1:** Themes of the factors (barriers and facilitators) influencing OP key behaviors, classified into TDF domains under COM-B components

Key behaviors	Themes of factors related to capability*	Themes of factors related to opportunity**	Themes of factors related to motivation***
Initiating the work modification process with the employee	Knowledge about e.g., why and how to modify work temporarily (C1) Skills to, e.g., evaluate the right timing of RTW (C2) Remembering to consider modified work during consultations (C3) Habits of reflecting on one’s work (C4)	Physical resources, e.g., time for initiating this option during consultation (O1) Experienced social pressure from different stakeholders (O2)	Conceptions of an OP role related to initiating the process (M1) Confidence in handling the process (M2) Strong personal aims related to SAW/RTW (M3) Beliefs about the consequences of being/not being an active initiator (M4) Feedback from earlier cases (M5) Feelings related to handling the process (M6)
Negotiating work modifications with the supervisor	Knowledge about the benefits of work modifications for companies (C1) Skills to negotiate with supervisors (C2)	Possibilities to modify work at companies (O1) Experienced social pressure from different stakeholders (O2)	Conceptions of an OP role related to activity with supervisors (M1) Beliefs about the consequences of being/not being active in cooperation with supervisors (M4)
Following up the work modification process	Remembering to follow up the process (C3)	Length of relationships with employees (O1)	Conceptions of an OP role related to following up the process (M1) Beliefs about the consequences of being/not being active in follow up (M4)

*Capability: *C1* Knowledge, *C2* Skills; *C3* Memory, attention and decision processes; *C4* Behavioural regulation

**Opportunity: *O1* Environmental context and resources, *O2* Social influences

***Motivation: *M1* Social/Professional role and identity, *M2* Beliefs about capabilities, *M3* Goals, *M4* Beliefs about consequences, *M5* Reinforcement, *M6* Emotion. (*M7* Optimism and *M8* Intentions were not relevant to our data)

In addition to describing the factors influencing the key behaviors, we identified OPs’ means to address the factors, e.g., by removing or diminishing barriers, and creating new enablers or enhancing the existing ones, in order to enable the desired behaviors. Tables 2, 3 and 4 present OPs’ experiences of and suggestions for means to target the identified factors.

**Table 2:** OPs’ experiences of and suggestions for means to increase Capability for using temporary work modifications (TWMs), with sample quotes

**Knowledge**
Knowledge has been acquired from:
Formal education (about why to use TWMs): *“From the lectures of this subject mainly, and then from the research evidence there is about it”. (OP5)*
Senior physicians and discussions with colleagues (about how to use TWMs): *“Introduction from a more experienced colleague is extremely important to get on as soon as possible” (OP12) “We pretty intensively go through each other’s cases also and ponder them together”. (OP13)*
Active practicing (about work at companies): *“I constantly try to get acquainted with different work possibilities and to find out what could be done there”. (OP14)*
**Skills**
Skills have been acquired from:
Active practicing (negotiation skills; right timing of RTW*)*: “*As one is kind of a veteran in this, and has been through these talks so many times, one develops a certain eye”. (OP10)*
Skills could be supported through:
Formal education (negotiation skills): *“Conversation and cooperation skills to make the patients realize why this would be in their own interest”. (OP3)*
Guidelines for evaluating the right timing of RTW: “*It would be nice to also have clear rules you can then lean on”. (OP4)*
**Memory, attention, and decision processes**
Remembering has been supported by:
Agreements between the occupational health provider and a company that TWMs are considered with all eligible employees: *“In one company we put a tremendous effort not to put people on sick leave unnecessarily before finding out, whether they have possibilities to return to their work with these limitations”. (OP3)*
Monitoring procedures predefined by the occupational health provider: *“In our occupational health center we have agreed upon a procedure that I myself also follow not to forget anything”. (OP12)*
**Behavioural regulation**
Behavioural regulation has been enhanced by:
Reflective discussions: *“Now (during the focus group discussion) I see what I then should have been able to use more and think about”. (OP4)*

**Table 3:** OPs’ experiences of and suggestions for means to increase Opportunity for using temporary work modifications (TWMs), with sample quotes

**Environmental context and resources**
Physical resources have been created/increased by the OPs:
Promoting agreements on TWMs procedures with companies: *“In our occupational health action plan we offer it, it’s an offer that is included in occupational health”. (OP8)*
Informing other colleagues about TWMs: *“We organized a meeting where we discussed this with GPs, and their role in it”. (OP12)*
Employer could provide OPs with more resources:
Possibility to focus on supporting SAW/RTW: “*Our work arrangements should be such that it’s possible to do this kind of work and not only medical treatment”. (OP12)*
Society-level means could be developed:
Easier procedure for applying benefits: *“I find the application process lousy. The Swedish model, for example, is more handy”. (OP5)*
Solutions for disadvantageous benefits: *“The systems should be such that it always is financially more advantageous to return to work”. (OP7)*
**Social influences**
Social resources have been increased by the occupational health provider:
Occupational health provider has informed companies about TWMs: *“Occupational health providers have conveyed the message about these support measures and why it’s profitable to favor them. The supervisors have understood the message”. (OP13)*
Social resources have been created/increased by the OPs:
Keeping TWMs short enough to promote coworkers’ positive attitudes: “*You can go on with this (TWMs) for a couple of months, but then you should reach more permanent solutions”. (OP4)*
Instructing supervisors on introducing TWMs to workplace: *“I have suggested that when a person returns to a work modified with easier tasks or shorter hours, there should be a meeting with the co-workers”. (OP14)*
Building trustful relationships with stakeholders: *“The trust … that we are not trying to deceive supervisors by smuggling disabled people to work”. (OP8)*
Informing union representatives: *“I try to inform the union representatives what all this is about”. (OP15)*
Social resources could be enhanced by society-level means:
Stakeholders’ attitudes towards TWMs should be influenced by social marketing: “*The companies and supervisors should be directly informed. Informing in public through many different channels”. (OP12)*

**Table 4:** OPs’ experiences of and suggestions for means to increase Motivation for using temporary work modifications (TWMs), with sample quotes

**Professional role and identity**
Proactive role can be developed through:
Instruction from senior doctors: *“I’d say that this role should pretty thoroughly be explained to the specializing doctors”(OP10)*
**Beliefs about capabilities**
Confidence in one’s capability to handle TWM processes has been developed through:
Active practicing among TWMs: *“At least I have the experience that you can modify a job. Sometimes it may be slow and take some time … And something may always go wrong but that’s life, isn’t it. You don’t have to worry about it”. (OP6 &OP8)*
**Beliefs about consequences**
Beliefs about the benefits of OP’s activity have been developed through:
Experiences from prior cases: *“People usually don’t even think that something could be done. The patients seldom come up with that idea (TWMs)”. (OP9) “When they are informed about this possibility, I can’t recall one person who hadn’t been enthusiastic about it and found it positive”. (OP13)*
**Goals**
Personal goals have been influenced by:
Societal-level messages concerning working life: “*The Finnish Institute of Occupational Health has been drumming us these ‘tidings of joy’:’no more long sick leaves and patients quickly back to work’. Every sick leave that I prescribe means a deduction to our national output”. (OP5)*
**Reinforcement**
Motivating feedback has been elicited through:
Reviewing the outcomes of one’s work: *“I just went through all my return-to-work negotiations for the last year. In most cases the outcome was a return to work and some kind of a positive solution”. (OP9)*
**Emotion**
No means mentioned to increase positive emotions

## Capability: Barriers and Facilitators and Physicians’ Means to Target Them

The following TDF domains are linked to Capability: Knowledge; Skills; Memory, attention and decision processes; and Behavioural regulation. All four domains were relevant to key OP behaviors. Physicians’ domain-specific means of targeting the identified barriers and facilitators are presented in Table 2.

Firstly, physicians may not even remember to consider SAW/RTW with temporary work modifications, if the employee does not bring it up her/himself (*Memory, attention and decision processes)*. Predefined agreements with companies that TWMs are routinely considered with all employees, and predefined procedures for following up the process enhanced remembering for some physicians.

Adequate *Knowledge* about TWMs and their benefits, including related legislation and benefit systems (e.g., part-time disability benefits) and about the work, working conditions and possible ways of modifying work in client companies were described as enablers of OP behaviors. Conversely, insufficient knowledge was mentioned as a hindering factor. Continuous education courses (formal education) and informal learning at the workplace (active practice among TWMs, learning from/with senior physicians and peers) were experienced as means to acquire knowledge.

Specialist training had not provided all physicians with sufficient *Skills* to evaluate the right timing for RTW and suitable work modifications. It was suggested that official guidelines for a proper length of sick leaves for specific (medical) conditions might facilitate OPs in decision making. However, some physicians had learned through repeated application to make ‘good enough’ evaluations. Having or lacking skills to negotiate with hesitant employees and/or supervisors was also discussed as a facilitator of or barrier to behaviors. Learning at work and continuous education courses were mentioned as means to gain adequate negotiation skills.

Learning new knowledge and practicing new skills may also be enhanced by reflecting on one’s actions and possible needs for change (B*ehavioural regulation)*. According to the physicians, discussions with colleagues in the focus group served as an arena for reflection.

The Behaviour Change Wheel (BCW) proposes that for addressing factors related to Capability, the following intervention functions be considered [16]: education, training, modeling, enablement, or environmental restructuring. Various behavior change techniques may be utilized to serve each intervention function. The means used or suggested by the participants are examples of these techniques. Continuous education courses and different types of informal learning at the workplace can be used to increase relevant knowledge and understanding (education), as well as to impart necessary skills (training). Guidelines for proper length of sick leaves, predefined agreements on TWMs with companies, and predefined follow-up procedures are examples of changing the physical or social environment in order to promote the desired behavior (environmental restructuring).

## Opportunity: Barriers and Facilitators and Physicians’ Means to Target Them

The following TDF domains are linked to Opportunity: Environmental context and resources; and Social influences. Both domains were relevant to key OP behaviors. Physicians’ domain-specific means of targeting the identified barriers to and facilitators of behaviors are presented in Table 3.

Physicians described different types of barriers and facilitators, related to *Environmental context and resources*. Available benefit systems (e.g., part-time sick leave in Finland) facilitate applying TWMs, although, according to participants, this option might be used more frequently if the medical certificate and the needed procedure was easier to complete. Part-time sick leave is financially disadvantageous for some employees, and may restrain physicians from offering this option. Societal-level solutions were considered necessary to target these barriers.

Physicians described that when there was poor cooperation within healthcare, TWMs is often no longer an option to employees. Instead of referring employees to OPs early enough, physicians (of other specialties) may prescribe overly long sick leaves. To overcome this barrier, some OPs had actively informed their GP colleagues about the possibilities of TWMs and collaboration with occupational health providers. However, employees themselves may postpone seeing a doctor until their work disability is too severe for RTW with temporarily modified work. To address this, companies can offer all employees regular screening for work ability problems, using occupational health services.

Insufficient time may hinder engaging in the TWMs process. In contrast, some occupational health providers were reported to facilitate OP behaviors by allowing them to organize own timetables and focus on supporting employees’ SAW/RTW instead of only treating medical conditions, and by organizing multi-professional support. Pre-established procedures, agreed with employers, can make work modification processes smoother. Some participants reported actively marketing these agreements to employers. Finally, following up the TWMs process is sometimes hindered, for example, when OP staff turnover occurs.

*Social influences* may originate from different stakeholders. Society was experienced to exert strong pressure on physicians to avoid long sick leaves and promote working life participation. Positive or negative social pressure from different stakeholders may encourage or inhibit engaging in the TWMs process. Informed and skilled supervisors were seen as important facilitators of OPs’ work. TWMs can be challenging to implement when supervisors expect OPs to prescribe sick leaves only or are reluctant to cooperate in general. Physicians perceived that supervisors’ resistance to work modifications was due to partiality and/or poor supervisory skills (e.g., caused by lack of experience, education or support from managers). In addition, company culture may not favor modified work. Furthermore, coworkers may resist modified work if relationships at the workplace are poor and/or trust in justice is low. Trade union representatives may perceive work modifications as a means of weakening employees’ rights.

However, some participants described means to influence workplace stakeholders’ attitudes and expectations. Work modifications need to be temporary enough to be acceptable for co-workers. Occupational health providers can distribute proper information about TWMs and their benefits to companies. Different stakeholders’ attitudes could be influenced on a large scale through social marketing. Some OPs had instructed supervisors on how to introduce modified work to their workplaces, and delivered proper information to union representatives. OP personally knowing the supervisors facilitated some contacts. To enhance this enabler, some OPs described paying special attention to nurturing mutual trust through frequent collaboration.

None of the OPs described means of persuading reluctant supervisors or employers when their resistance was seen to arise from the following factors: compared to big companies, companies with limited range of duties have few possibilities to modify work; small companies have less financial incentives to support SAW/RTW; supervisors are sometimes overly burdened by already large demands for modified work; and work life is hectic in many companies, due to national recession.

The BCW framework proposes that the following intervention functions be considered for targeting the Opportunity-related determinants of behavior: training, modeling, enablement, environmental restructuring, or restriction. Our participants described how the application of TWMs has been or could be further enhanced by providing OPs with adequate physical and social resources. In addition, some OPs had already actively strived to change the physical or social context by themselves. These means, along with the suggested society-level actions, are examples of the behavior change strategies serving the environmental restructuring and enablement functions.

## Motivation: Barriers and Facilitators and Physicians’ Means to Target Them

The following TDF domains are linked to Motivation: Social/professional role and identity; Beliefs about capabilities, Optimism; Beliefs about consequences, Intentions; Goals; Reinforcement; and Emotion. All domains apart from Optimism and Intentions were relevant to the key OP behaviors. Intentions, i.e. ‘conscious decision to perform a behavior or a resolve to act in a certain way’ [20], was not relevant, because all participants already used TWMs at least occasionally. No data were deemed to match Optimism, which refers to a general disposition rather than specific capabilities (‘the confidence that things will happen for the best or that desired goals will be attained’). Physicians’ domain-specific means of targeting the identified barriers and facilitators of behavior are presented in Table 4.

Experiences of successful cases (*Reinforcement)* as well as finding the related behaviors personally satisfying or enjoyable (*Emotion)* may motivate physicians to initiate and work through the TWMs process. Encouraging feedback can be elicited through reviewing the outcomes of one’s work. Strong personal *Goals*, related to enhancing work ability and work participation among all employees, promote active behaviors in this practice, and can be stimulated through public debate.

Participants expressed divergent views of an OP’s role with regard to using TWMs (*Professional role*). We formulated a continuum of an OP role, with the end points labeled as “reactive role” and “proactive role”. A more reactive role refers to engaging in this practice mostly when it is suggested by the employees or supervisors. Serving as a medical expert is emphasized as the core of the role at the expense of other duties. In contrast, physicians advocating a more proactive OP role perceived themselves as responsible for bringing up this option with all eligible employees. Applying TWMs was considered to match well with their cooperative role of serving both employees and employers. On the whole, these physicians emphasized supporting employees’ workability by diverse means instead of only treating medical conditions. A proper orientation to the multifaceted work, provided by senior colleagues, was seen to support professional growth into a more proactive role.

In addition to having divergent conceptions of a proper OP role, physicians’ actions were influenced by their *Beliefs about consequences*. Some participants believed that active recommending and planning TWMs and monitoring the process is beneficial for most employees and supervisors. Experiences at work had shaped these beliefs. In contrast, believing that a physicians’ intervention is not generally necessary, or that TWMs may affect an employee negatively may hinder introducing this option. In general, positive B*eliefs about one’s capabilities to* handle even tricky TWMs processes, can fuel activity among this practice. These beliefs had developed through active practicing among TWMs.

The BCW framework proposes that for addressing factors related to Motivation, the following intervention functions be considered: education, training, modeling, persuasion, enablement, environmental restructuring, incentivization, or coercion. In our data, instruction provided by senior physicians is an example of the techniques serving the educational function. Active practicing among TWMs and conscious reviewing of the outcomes of one’s work can have training and enabling functions. Strong societal messages concerning work participation, delivered through public debate, is an example of a behavior change technique serving persuasion.

## Discussion

The three key behaviors that OPs engage in when applying TWMs to support SAW/RTW were initiating the process during consultation with the employee; making recommendations to the workplace; and monitoring the work modification process. The BCW and the TDF were used to identify factors (barriers and facilitators) that influence the key OP behaviors and could be targeted in future interventions.

Application of TWMs may be influenced by factors related to physicians’ capability. Having adequate knowledge and skills of the “what, why, when and how” of the TWMs were discussed as impacting behavior. In addition, the level of routinization with regard to applying TWMs, as well as reflecting on one’s work and its outcomes, were identified as factors related to individual capability.

Physicians’ motivation to apply TWMs may be influenced by their beliefs regarding personal capability and the consequences of their actions, and by more general goals, prior experiences and emotions related to this practice. In addition, participants described divergent conceptions of a proper OP role and related behaviors.

Various factors related to physical and social environment (opportunity) may influence physicians’ behavior. Physical resources afforded by the occupational health provider (e.g., time, multi-professional support) and client companies (e.g., availability of modifiable duties, predefined TWMs procedures) were presented to impact OP behavior. Experienced social pressure from different stakeholders was also discussed in the interviews.

Our findings expand previous understanding of the relevant knowledge, skills and attitudes needed from occupational health practitioners, as well as the multi-level environmental factors that influence the application of work modifications [11–15, 35–38]. By utilizing the TDF we were able to conduct a comprehensive theory-informed assessment of all potential factors that influence OPs’ key behaviors. Categorization of these factors into the three components of behavior (capability, opportunity and motivation) clarified the analysis of what needs to change in order to promote physicians’ activity in using TWMs.

Unlike previous RTW-related studies, we utilized a theoretical framework, the BCW, to inform the development of future interventions. We evaluated the possible applicability of the intervention functions proposed by the BCW by investigating physicians’ perceived means of overcoming the barriers and/or enhancing the enablers. Our results suggest that at least five intervention functions be considered when designing future interventions: education, training, persuasion, environmental restructuring, and enablement.

Formal education (e.g., CME courses) might be useful for providing physicians with evidence-based knowledge and understanding about SAW/RTW and TWMs, and opportunities for training relevant skills. However, further education may be perceived as unnecessary if needs for learning are not recognized.

Informal learning at the workplace is effective for acquiring relevant knowledge and skills, for updating beliefs about one’s capability and consequences of actions, and for developing one’s conceptions of an OP role. Workplace learning can include learning from/with senior physicians and peers, as well as learning through experiences gained in applying TWMs. Reflection on one’s work and its outcomes supports efficient learning from experiences [39, 40]. Yet, reflective practice may not be familiar to all physicians, and could be instructed in formal education [41].

Consistent with previous studies, our results indicate that in order to promote physicians’ activity in applying TWMs, changes might be needed not only at the intra-physician level, but at organizational and society levels also. According to participants, occupational health services could promote the use of TWMs by providing OPs with better physical and/or social resources, such as the possibility to focus on supporting SAW/RTW, and informing employers about this opportunity. Society-level actions were also suggested by our participants, such as creating incentives for employees and employers to use TWMs, providing physicians with guidelines for proper length of sick leaves and more usable procedures for applying benefits, and delivering proper information about the possibilities and benefits of SAW/RTW via TWMs to all stakeholders on a large scale.

The first key behavior, initiating TWMs with an employee during consultation is fundamental to the process. Most barriers and enablers that were identified were related to this key behavior. Some physicians had actively acquired relevant capabilities through frequent use of TWMs and created more physical or social resources by, for example, striving to influence different stakeholders’ attitudes towards TWMs. Our results suggest that targeting the motivation component of behavior, and the conception of one’s professional role especially, may be important in interventions to promote the use of TWMs. It seems that among our participants, the adoption of a proactive OP role generated actions which in turn increased supportive knowledge, skills and beliefs, provided rewarding experiences, and consequently, boosted self-confidence in applying TWMs.

## Strengths and Limitations of the Study

This is the first study to our knowledge to use the frameworks of TDF and BCW in a return-to-work context. They provided both a comprehensive assessment of the factors that are likely to influence the key OP behaviors and informed the development of future interventions to promote the desired behaviors. In many countries physicians have the task to assess the work ability of their patients and to suggest possible interventions to enhance return to work. We believe that the utilization of the TDF and the BCW might be helpful in identifying the barriers and facilitators of this practice in a variety of jurisdictions and contexts, although the specific factors and intervention functions may be context-specific.

Our results are based on physicians’ accounts of their behaviors, and may not represent their actual practice. Furthermore, some of the perceptions about barriers and facilitators may be beliefs, not based on authentic experiences. However, physicians’ accounts of the facilitating and hindering factors related to other stakeholders’ attitudes and actions, despite the Finnish context, coincide with the results from previous RTW-related studies. Employers or supervisors may be cooperative or reluctant to work modifications [42–44]. Employees themselves may approve or refuse work modifications for different reasons [45, 46]. Work modifications often influence coworkers, who may be more or less willing to support the returning employee [47–49]. Future studies on the experiences and opinions of different workplace stakeholders with regard to RTW and work modifications might benefit from using theoretical frameworks, such as the TDF and the BCW.

Observations of physicians engaging in this practice with employees and employers would contribute to our understanding of this multifaceted process involving various actions with different stakeholders. In addition, a quantitative study would be needed to determine the frequency of this practice among Finnish OPs and the importance of and the inter-relationships of the determinants of behavior.

Because the physicians in our study volunteered to participate, they may represent a subgroup of OPs who have a higher degree of interest in supporting SAW/RTW compared to other OPs. However, the results show that the participants varied with regard to their self-evaluated capability and motivation to use TWMs. Larger focus groups might have produced a more extensive variety of OPs’ experiences and reasoning. However, small groups allow for active participation of all members and in-depth discussion on the topic.

We consider our sample to be adequate, as it generated rich data and produced new insights into return to work, all the while within the context of a difficult-to-access community. We believe that further studies with more physicians would be useful and that this study provides a good starting point for theoretical development.

Studies applying the TDF have often gathered their data using an interview topic guide specifically designed to capture the TDF domains [19, 20]. The domains of the TDF were not considered when we designed the pilot interview topic guide and the focus group propositions. Although the TDF did not guide the data collection of our study, we were able to carry out the data analysis using it as a coding framework. In addition, we may have gathered richer data by allowing unanticipated issues to emerge during the interviews and group discussions because the interview topics and propositions were not restricted by the TDF.

## Conclusions

Our study illustrates how the TDF and the BCW can be applied in the RTW context to investigate which determinants of physicians’ behavior need to be targeted, and how, to promote desired behaviors. The specific intervention strategies and modes of delivery need to be determined depending on the context.

## Electronic supplementary material

Below is the link to the electronic supplementary material.

Supplementary material 1 (PDF 419 KB)
