# Supplementary material for: Application of the Theoretical Domains Framework and the Behaviour Change Wheel to Understand Physicians’ Behaviors and Behavior Change in Using Temporary Work Modifications for Return to Work: A Qualitative Study
Source: J Occup Rehabil. 2017 Apr 8;28(1):135–46. doi: 10.1007/s10926-017-9706-1 (PMC5820403; doi:10.1007/s10926-017-9706-1)
Supplement: Supplementary file 1 — Supplementary material 1 (PDF 419 KB) [file 10926_2017_9706_MOESM1_ESM.pdf]

Appendix A

Application of Theoretical Domains Framework and Behaviour Change Wheel to understand physicians' behaviors and behavior change in using temporary work modifications for return to work

Journal of Occupational Rehabilitation

Horppu R, Martimo KP, MacEachen E, Lallukka T, Viikari-Juntura E

Ritva Horppu, PhD, Corresponding author

Work Disability Prevention, Finnish Institute of Occupational Health, Helsinki

GSM: +358 46 8505129, E-mail: ritva.horppu@ttl.fi

Appendix A. Summary of facilitators of and barriers to key OP behaviors, classified into TDF domains under COM-B components, with sample quotes

**CAPABILITY**
**KNOWLEDGE** (An awareness of the existence of something, e.g., knowledge of scientific rationale; procedural knowledge; knowledge of the environment)<br><br>**Facilitators:**<br>OPs have knowledge about:<br>• Benefits of temporary work modifications (TWMs) for different stakeholders: *”There is research evidence that the longer the sick leave the more difficult to return to work. It makes sense to work shorter hours when a person is motivated to work”. (OP12)*<br>• Work, working conditions and possibilities to modify work in the companies: *”As occupational physicians we have the advantage of knowing the company, its situation and often these employees, their previous medical history and background”. (OP14)*<br>• Legislation and benefit systems (e.g., part-time sick leave) related to TWMs: ”*I think that the supervisors may not always be aware of these new possibilities. They pretty well know about the partial sick leave but not always the work trial and other stuff that could be done. I’m used to inform them about these options when calling them”. (OP12)*<br><br>**Barriers:**<br>OPs lack knowledge about:<br>• Benefits of TMWs for employers: ”*I think that all doctors command pretty well the medical diagnostics and treatment. But then this other stuff: how could you, for example, make the supervisor understand how the employer could benefit from the effort of shortening peoples’ unnecessary sick leaves”. (OP3)*<br>• Work, working conditions and possibilities to modify work in the companies: *”There are many companies with which we are not in contact very often, because we may not realize the potential problems and the workplace visit may have been conducted before the existence of this new system”. (OP12)*
**SKILLS** (An ability or proficiency acquired through practice)<br><br>**Facilitators:**<br>OPs have skills related to:<br>• Evaluating the right timing for safe RTW and suitable work modifications for employees: ”*Certainly you´d have to diagnose the disease, and somehow estimate the degree of recovery. And then knowledge about the job whatever it might be. And also some kind of personal touch. I then try to sense the patients’ readiness for return to work by discussing with them.” (OP10)*<br>• Negotiating with hesitant employees/supervisors: *”I always challenge them (supervisors) by asking “C’mon, how often you yourself continuously work with a 100% efficiency? You surely can’t expect that from your subordinates, that’s quite impossible.” (OP6)*<br>• Cooperating with employees and supervisors: *”We don’t really have other ways to bring about the work accommodations but in cooperation, based on what the patient and the employer think.. .. You have to reach a mutual agreement. You can’t advocate the work modifications as if you were in court”. (OP10 & OP11)*

Barriers:

OPs lack skills related to:

- Evaluating the right timing for safe RTW and suitable work modifications for employees: *"It's terribly hard for everybody if attempts to return to work fail. But how can you identify that this person might be able to manage at work if the tasks are modified a little? It seems to me that we don't have suitable indicators for judging with whom it (RTW) might succeed". (OP4)*
- Negotiating with hesitant employees and/or supervisors: *"And interactions with the employers. It's pretty strange to doctors how they think and how they should be approached. They are interested in business. Somehow the communication still is somewhat awkward and difficult". (OP4)*

MEMORY, ATTENTION AND DECISION PROCESSES (The ability to retain information, focus selectively on aspects of the environment and choose between two or more alternatives)

Facilitators:

- OPs remember to consider the possibility of TWMs during consultation: *"I hope that this (TWMs) will be used increasingly and on a more routine basis in our clinics. It's almost regular or daily work for me to evaluate the ability to work and function and I also guide others to do this". (OP14).*
- OPs routinely follow up the work modification process: *"During the negotiation of a specific employee we make an agreement on the contacts for following up: when, by whom and to whom" (OP12)*

Barriers:

- OPs do not remember to consider the possibility of SAW/RTW via TWMs during consultations: *"One may not always remember that this option (TWMs) exists. This may happen to others as well". (OP4)*

BEHAVIOURAL REGULATION (Anything aimed at managing or changing objectively observed or measured actions)

Facilitators:

- OPs are accustomed to reflective practice: "*Thinking about some of my own actions at the time I started working as occupational physician I feel almost terrified. Today I wouldn't act the way I did then. We just need to remember to educate those entering this field and help them in these situations" (OP7)*

OPPORTUNITY

ENVIRONMENTAL CONTEXT AND RESOURCES (Any circumstance of a person's situation or environment that discourages or encourages the development of skills and abilities, independence, social competence, and adaptive behavior)

Facilitators:

Several factors facilitate OPs in applying TWMs to support SAW/RTW, including:

- Societal regulations and benefit systems: *"More tools have been developed during the years, this kind of down-to-earth tools, that I find good". (OP10).*
- Predefined procedures for TWMs agreed between the occupational health provider and companies: *"It has been agreed in the yearly action plan meetings that we utilize this arrangement and that it concerns everybody". (OP14)*
- Able supervisors: *"Supervisors have a demanding task to notice and discover the work ability problems, and to know how they can contact the occupational health service. In our*

model of early intervention we have a read-made model for educating supervisors, and the occupational psychologist can train them in three hours". (OP10)

- Time resources and multi-professional support provided by the occupational health provider: *"You cannot know yourself all that could be supportive for the work ability of a particular person, for example, with regard to musculoskeletal problems. That's why I utilize so often the physiotherapists, because I think they may be better able to advise (the patients)". (OP15) "This (applying TWMs) works pretty well at least in my job, because I am able to allocate quite a bit of my time especially to those who have problems with coping at work". (OP13)*
- Good cooperation within healthcare: *"I have been delighted when I've had, for example, some cases with musculoskeletal problems, and even an orthopedist has recommended this (TWMs)". (OP13)*
- Regular screening of work ability problems among all employees: *"These work ability surveys for all employees are quite good" (OP6)*

Barriers

Several factors may hinder OPs in applying TWMs to support SAW/RTW, including:

- Disadvantageous benefit systems for employees: *"If an employee earns more money by being on sick leave than by returning to work part-time, it's awfully difficult to motivate most people to do that". (OP3)*
- Poor usability of procedures needed for arranging RTW via TWMs: *"I have applied partial sick leave even with the minimum length, 12 days, but it involves an unreasonable amount of bureaucracy and paperwork. (OP7)*
- Limited possibilities or financial disadvantages to modify work in (small) companies: "*Very small companies do not always have possibilities to modify work. If there is only some kind of heavy physical labor available, and an employee has a physical impairment, this (TWMs) doesn't function there". (OP14)*
- Hectic working life in companies in general: *"Working pace and work standards have become tighter all the time. It seems to me that during the 70s' and 80s' it was easier to modify work for example for cleaners by diminishing the area to be cleaned or something similar. But now it seems to me that everything has been pulled so tight. It (TWM) is more difficult now". (OP9)*
- Unable supervisors: "*Supervisors differ with regard to their general supervisory skills". (OP14)*
- Poor cooperation within healthcare: *"There are some colleagues who may think that work is very dangerous. Psychiatrists easily prescribe sick leaves for many months, and then we are quite confused". (OP6)*
- Employees postponing their visits to occupational health: *"A big problem is the people who too late become customers of the healthcare system in general. We cannot make many temporary work accommodations for them anymore. Impairments have already become permanent". (OP6)*
- Poor time resources provided by the employer: *"As we are expected to consider in each case of sick leave if these various modifications could be applied, in my opinion, the healthcare system should allocate time for us to evaluate the situation of each particular person." (OP7)*
- Following up hindered if OP in charge resigns his/her position: *"I left to specialize here (at FIOH), so I didn't see if it (TWM) was ultimately successful, but it looked pretty good." (OP4)*

SOCIAL INFLUENCES (Those interpersonal processes that can cause individuals to change their thoughts, feelings, or behaviors)

Facilitators:
Social pressure may be exerted on OPs to initiate TWMs by:

- Employees: *"Actually, I've lately encountered more situations where people have been prescribed long sick leaves and they have themselves consulted me to ask if they could return to work. They come to ask for a permission to do so". (OP14)*
- Supervisors/employers: *Some employers already know how to contact me and ask if it (TWM) could be considered for an employee. When these (TWMs) have become more common, employers may remember how well it worked with in a particular case and 'could it be considered with this one as well'. (OP2)*
- Society: *"There is a strong pressure on occupational physicians related to sick leaves. This message has come across well". (OP5)*

Barriers:
Social pressure may be exerted on OPs NOT to initiate TWMs by:

- Employees: *"There still are people who believe they can't go to work when they need one single pill of painkiller, because that means they are not well". (OP15)*
- Supervisors/employers: "*When there is a multi-skilled employee at the workplace, it may easily be said 'as soon as she/he comes to work, we'll definitely find work for her/him to do'. And then sometimes you notice that there are people who are not wanted at their workplaces. Then these work modifications are awfully difficult to do". (OP3)*
- Co-workers: *"It's probably field-specific and may depend much on the workplace, but I believe that in cases of hard manual labor work modifications can be considered as an easy way out of the hard tasks. This may evoke envy". (OP12)*
- Union representatives: *"Sometimes the workers' union or its representatives may think that 'our people must be allowed to be sick in peace'. They miss the point of view of the work having a rehabilitative role. In a way their excessive protective measures turn against the best interests of an employee". (OP7)*

MOTIVATION

SOCIAL/PROFESSIONAL ROLE AND IDENTITY (A coherent set of behaviors and displayed personal qualities of an individual in a social or work setting)

Facilitators:

- Adoption of a proactive OP role: *"When I started working in occupational health, I feel like I experienced an enlightenment, that this is not only about medical treatment but also involves all kinds of other stuff. That this involves these three: the employee, the work community and the work environment. I realized the possibilities, how different from plain medical treatment my work can be." (OP12)*

Barriers:

- Adoption of a more reactive OP role: *"They may perceive their roles as being more doctors treating patients not wanting employers to know anything about the situation. They may think that this is no part of the duties of an occupational physician. They may be busy with much work to do, and all this takes time, and so on". (OP14)*

BELIEFS ABOUT CAPABILITIES (Acceptance of the truth, reality, or validity about an ability, talent, or facility that a person can put to constructive use)

Facilitators:

- Having faith in one's ability to use TWMs: *"Most of the negotiations, probably 95%, proceed in full understanding, and everybody wants the same outcome. Then you just*

ponder how you can achieve that. The conflict situations are the ones you just have to try to navigate through somehow". (OP9)
**BELIEFS ABOUT CONSEQUENCES** (Acceptance of the truth, reality, or validity about outcomes of a behavior in a given situation)<br><br>**Facilitators:**<br>• Beliefs about positive consequences for employees/supervisors: *"The employee is really grateful when he/she can stay at work and doesn't have to be on sick leave unnecessarily. And the supervisor, on his behalf, is pleased that a problem was so surprisingly easily solved". (OP3)*<br>• Beliefs that personal involvement is needed or valuable in initiating and/or managing the TWM process: *"With musculoskeletal disorders it's easier to figure out in the workplace that 'a higher or lower chair is needed'. But when it comes to the mental health problems, they are more intimate and more difficult to discuss among themselves. It is beneficial if the physician figures out what the restraint is and then makes a suggestion straight to the employer how this particular work could be rearranged". (OP4)*<br><br>**Barriers:**<br>• Beliefs about negative consequences for an employee: *"Some patients mention that there is some kind of negative atmosphere when they return to work". (OP13)*<br>• Beliefs that personal involvement is NOT needed or valuable in initiating and/or managing the TWM process: *"When they decide themselves about it (work modification) at the workplace, it can often be more easily carried out. Often if it comes from a doctor's initiative, it may go all wrong, especially if the doctor does not know the workplace conditions". (OP1)*
**REINFORCEMENT** (Increasing the probability of a response by arranging a dependent relationship, or contingency between the response and a given stimuli)<br><br>**Facilitators:**<br>• Successful prior cases motivate OPs to carry on with this practice: *"It's probably the successful cases". (OP3)*
**GOALS** (Mental representations of outcomes or end states that an individual wants to achieve)<br><br>**Facilitators:**<br>• OPs have personal goals to avoid prolonging sick leaves and/or promoting working life participation: *"When you think about how much you can influence somebody's life by discovering supportive means and lengthening his/her working life through them, it really is a remarkable matter. That's why these are the things you should take seriously and with respect rather than by pushing the person just somewhere to get him/her out of the hands". (OP12 & OP13)*
**EMOTION** (A complex reaction pattern, involving experiental, behavioral, and physiological elements, by which the individual attempts to deal with a personally significant matter or event)<br><br>**Facilitators:**<br>• OPs enjoy their duties around TWMs: *"It's nice to be able to work on this (TWMs), this kind of work suits me well. I enjoy very much all the situations with employers and patients". (OP5)*

**Barriers:**<br>• Negotiations with employers may be unpleasant: *"Employers are very determined, they may say to you 'now listen to me miss, girl, or nowadays even lady, you have no idea how much all this is going to cost us'. (OP4)*
**OPTIMISM** (The confidence that things will happen for the best or that desired goals will be attained).<br><br>Not relevant for key OP behaviors
**INTENTIONS** (A conscious decision to perform a behavior or a resolve to act in a certain way)<br><br>Not relevant for key OP behaviors
